# Preclinical Activity of Embryonic Annexin A2-Specific Chimeric Antigen Receptor T Cells Against Ovarian Cancer

**DOI:** 10.3390/ijms21020381

**Published:** 2020-01-07

**Authors:** Leonard Leong, Heng Liang Tan, Simeon Cua, Kylie Su Mei Yong, Qingfeng Chen, Andre Choo

**Affiliations:** 1 Bioprocessing Technology Institute (BTI), Agency for Science, Technology and Research (A*STAR), Singapore 138668, Singapore; 2 NUS Graduate School for Integrative Sciences and Engineering (NGS), National University of Singapore, Singapore 119077, Singapore; 3 Institute of Molecular and Cell Biology (IMCB), Agency for Science, Technology and Research (A*STAR), Singapore 138673, Singapore; 4 Department of Physiology, Yong Loo Lin School of Medicine, National University of Singapore, Singapore 117545, Singapore; 5 Department of Biomedical Engineering, Faculty of Engineering, National University of Singapore (NUS), Singapore 117575, Singapore

**Keywords:** CAR-T cells, Ovarian cancer, Annexin A2, Solid tumours

## Abstract

Chimeric antigen receptors (CARs) have found clinical success in B cell malignancies, but a dearth of potential targets limits their wider clinical application, especially in solid tumours. Here, we describe the development of an anti-annexin A2 CAR, CAR(2448), derived from an antibody found to have activity against epithelial ovarian cancer cell lines. The spacer length of CAR(2448) was optimised based on in vitro cytotoxic activity against ovarian cancer (OC) cell lines via a real-time cytotoxicity assay. The longer spacer CAR(2448)L T cells exhibit significant effector activity, inducing inflammatory cytokine release and cytotoxicity against OC cell lines. Furthermore, CAR(2448)L-BBz T cells induced enhanced survival in an in vivo OC xenograft model and reduced tumour volume by 76.6%. Our preclinical studies of CAR(2448) suggest its potential for the unmet need of novel strategies for the treatment of ovarian cancer.

## 1. Introduction

CAR T cells have found success in clinical practice recently, with the FDA approval of tisagenlecleucel [1] and axicabtagene ciloleucel [2] against CD19 on B cell leukaemias and lymphomas. The success of anti-CD19 CAR T cells has paved the way for the use of CAR T cells against a range of other malignancies.

While CAR T cell have proven to be effective therapeutic agents in even aggressive haematological malignancies, their current application in solid tumours is limited. A number of factors cause the tumour microenvironment to be hostile to CAR T cells. These include immune checkpoint molecules such as PD-L1 that induce T cell exhaustion, infiltrating regulatory T cells and myeloid-derived suppressor cells that negate CAR T cell toxicity, and a dense extracellular matrix that inhibits T cell extravasation into the tumour space. Furthermore, a dearth of antigen targets prevents CAR T cells from being more widely applied for the treatment of cancer [3,4,5].

A particular class of targets that has had limited exploration in CAR T cells against solid tumours are glycoepitopes. Aberrant glycosylation in cancer can be induced by the dysregulation of glycosyltransferases, altering both the function and molecular profile of tumour cells [6]. Hence, CAR T cells that target glycans can potentially reduce on-target, off-tumour side effects, as a result of differential glycosylation between normal cell and tumour cells. However, only a limited number of anti-glycan CAR T cells targets have been explored clinically to date, including TAG72 in colorectal cancer, Lewis Y in acute myeloid leukaemia, and GD2 in neuroblastoma [7].

Epithelial ovarian cancer has the highest mortality rate among gynaecological malignancies [8]. The standard line of treatment for ovarian cancer involves surgical debulking, before platinum and taxane chemotherapy, however, due to the late-stage detection of most cases of ovarian cancer, long-term survival rates among patients remain poor [9]. In recent years, a few targeted therapies have entered clinical practice for the treatment of ovarian cancer VEGF-A antagonists as anti-angiogenic agents, and PARP inhibitors in BRCA1 and BRCA2 mutants [10]. However, the majority of ovarian cancer patients still lack targeted therapies that are capable of controlling their disease.

Previously, Cua et al. reported on a monoclonal antibody (mAb), mAb 2448, which targets an N-linked glycoepitope of annexin A2 (ANXA2). mAb 2448 is an IgG1 which is internalised into target cells and can function well as an antibody-drug conjugate. mAb 2448 exhibits binding to ovarian and breast cancer cell lines with an epithelial phenotype. In addition, mAb 2448 exhibits antibody-dependent cell-mediated cytotoxicity as a naked antibody in a xenograph model against ovarian cancer [11].

As ANXA2 plays a known role in the epithelial-mesenchymal transition (EMT) [12], targeting ANXA2 has potential as a therapeutic approach [13]. ANXA2 has been detected by immunohistochemistry in ovarian and breast cancer [14,15]. Surface-expressed ANXA2 mediates extracellular matrix degradation and neovascularisation via the production of plasmin, and has been found to correlate with invasion and metastasis in a range of cancers, including breast cancer, pancreatic ductal adenocarcinoma, renal cell carcinoma, colorectal cancer, and nasopharyngeal cancer [16]. In particular, ANXA2 overexpression correlates with poor prognosis in ovarian and breast cancer [17]. These features suggest the potential of annexin A2 as a target for immunotherapy.

Here, we report on CAR(2448), a CAR derived from the scFv of mAb 2448. CAR(2448) induces cytotoxicity and cytokine release upon recognition of target ovarian tumour cells expressing annexin A2 in vitro, and mediates tumour clearance in a xenograft murine model.

## 2. Results

### 2.1. Long Spacer CAR(2448) Mediates Optimal Cytotoxicity

CAR T cell function is dependent on the ability of CAR T cells to recognise target cells and induce signal transduction that activates the T cells to mediate its cytotoxic effect against the target cells. The formation of an immune synapse must exclude molecules such as CD45, which can inhibit CAR phosphorylation and downstream T cell activation [18,19]. In order to maintain an optimal immune synapse length, the spacer region of the CAR construct has to be tailored to the proximity of the target antigen to the membrane surface [20].

**Figure 1:**
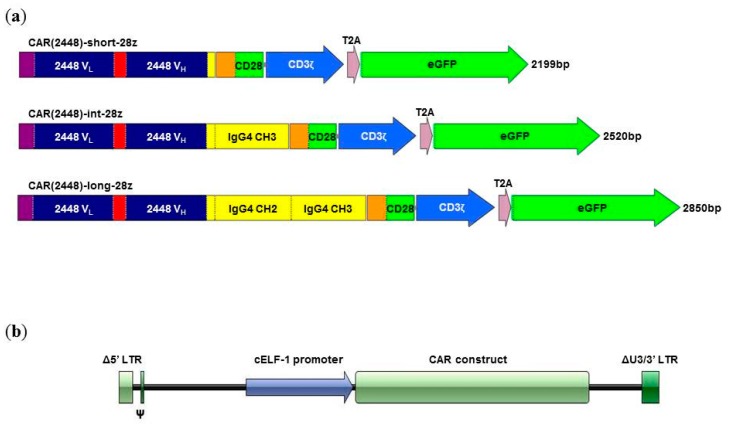
Construction of CAR(2448). (**a**) Spacer length variants of CAR(2448) created via truncation of the IgG4 hinge-CH2-CH3 domains. CAR(2448) is a second generation CAR consisting of the scFv of mAb 2448, a spacer domain, the CD28 transmembrane and signalling domain, and the CD3ζ domain in sequence. The short spacer consisted of the IgG4 hinge domain (12 aa), the intermediate spacer contained the IgG4 hinge-CH3 domains (122 aa), while the long spacer included the IgG4 hinge-CH2-CH3 domains (229 aa). A reporter gene (eGFP) was inserted downstream of the CAR construct via the T2A peptide. (**b**) Schematic of lentiviral vectors utilised for CAR transduction.

Tuning of the immune synapse formed between the CAR T cell and target cell has to be optimised for a novel CAR construct. To evaluate the optimal spacer for CAR(2448), mRNA vectors corresponding to short [CAR(2448)-short-28z], intermediate [CAR(2448)-int-28z], and long [CAR(2448)-long-28z] spacer length CARs were synthesised via in vitro transcription (Figure 1a). The long spacer, which contains the IgG4 CH2 domain, was modified to remove FcγR binding residues.

T cells were isolated from thawed PBMCs (StemCell Technology, Vancouver, BC, Canada), and activated for 48 h with anti-CD3/CD28 Dynabeads before nucleofection. T cells were nucleofected with equimolar concentrations of CAR(2448) spacer variant mRNA, resulting in high levels of T cell surface CAR expression (85.6 ± 2.7%) (Figure S1a). CAR surface expression on T cell surface peaked at 12 h post-transduction (6.5 geometric mean ratio compared to control), before decreasing, falling four-fold by 48 h post-nucleofection (1.6 geometric mean ratio) (Figure S1b). Hence, all assays utilising mRNA nucleofected T cells were carried out within 12 h of nucleofection, ensuring high CAR expression during co-incubation.

IGROV-1 and SKOV3 are ovarian cancer cell lines which have high expression of ANXA2 [11], corresponding to highly antigen positive, and partially antigen positive ovarian malignancy populations (Figure S2a). This was also validated by western blot (Figure S2b). As such, these cell lines were hence utilised as target cells to determine the functional activity of CAR(2448) T cells.

**Figure 2:**
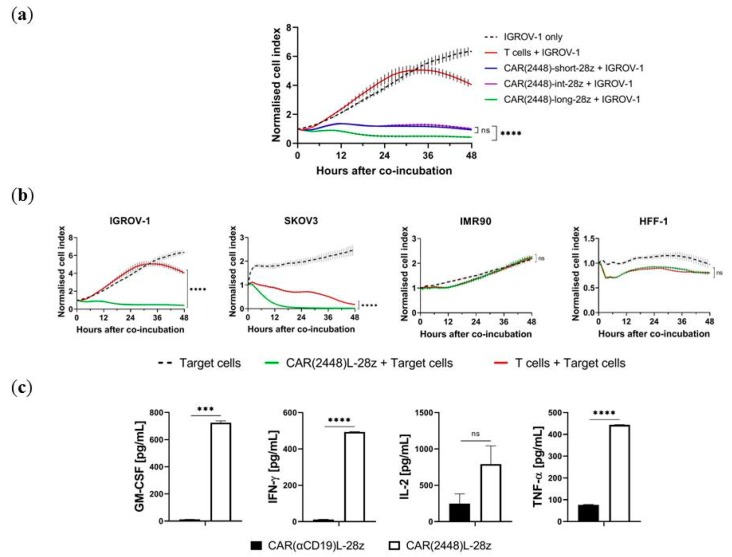
mRNA transfected chimeric antigen receptors (CAR)(2448) T cells mediate cytotoxicity. (**a**) CAR(2448)-long-28z T cells mediate superior cytotoxicity against IGROV-1 cells compared to CAR(2448)-short-28z or CAR(2448)-int-28z T cells. (**b**) CAR(2448)L-28z T cells mediate cytotoxicity against target cells expressing annexin A2 (IGROV-1 and SKOV-3), but not control cell lines (HFF-1 and IMR90). Co-culture conducted at 20:1 effector-to-target (E:T) ratio. (**c**) Co-incubation of CAR T cells with target IGROV-1 cells at a 10:1 E:T ratio induces inflammatory cytokine release for CAR(2448)L but not CAR(αCD19)L. For all subfigures, abbreviations: ns not significant. *** *p* < 0.001. **** *p* < 0.0001.

CAR(2448) T cells of variant spacer lengths were co-incubated with IGROV-1 cells at a 20:1 effector-to-target (E:T) ratio in an impedance-based real-time growth monitoring assay. While all CAR(2448) spacer variants mediated cytotoxicity against target cells, the kinetics of target cell clearance was dependent on the spacer length (Figure 2a). In particular, CAR(2448)-long-28z mediated the most efficacious cytotoxicity against target cell lines compared to either CAR(2448)-short-28z (*p* < 0.0001) or CAR(2448)-int-28z (*p* < 0.0001) spacer CAR(2448) constructs. Similar results were found against SKOV3 (Figure S3). This is consistent with the membrane proximal position of ANXA2 on the surface of tumour cells [16]. Based on this cytotoxicity data, all subsequent experiments were carried out using the long spacer CAR(2448), CAR(2448)L.

### 2.2. mRNA Vector CAR(2448) T Cells Exhibit Anti-Tumour Activity Against ANXA2+ Ovarian Cancer Cells

In order to further validate CAR(2448) before derivation of the long-term expression lentiviral model, CAR(2448)L T cells were co-incubated with target ANXA2-positive (ANXA2+) cells lines (IGROV-1 and SKOV3), or with ANXA2-negative (ANXA2-) normal cell lines (IMR90 and HFF-1) (Figure S2). IMR90 is a normal human lung fibroblast cell line, while HFF-1 is a human foreskin fibroblast cell line. Target cell growth was monitored in real-time.

CAR(2448)L-28z mediated targeted cell killing was only observed against ANXA2+ target cells. CAR(2448)L T cells effectively killed IGROV-1 (*p* < 0.0001) and SKOV3 (*p* < 0.0001), but did not induce cytotoxicity against ANXA2- control cells lines, IMR90 (*p* > 0.9999) and HFF-1 (*p* > 0.9999) above the level of control T cells (Figure 2b).

In addition, when co-incubated with target IGROV-1 cells, CAR(2448)L-28z T cells mediated significant levels of inflammatory cytokine secretion as compared to T cells nucleofected with the control CAR(αCD19)L-28z, including GM-CSF (*p* < 0.001), IFN-γ (*p* < 0.0001), and TNF-α (*p* < 0.0001) (Figure 2c).

### 2.3. Lentivirally Transduced CAR(2448) T Cells Mediate Cytotoxicity and Cytokine Release Against ANXA2+ Ovarian Cancer Cells

While mRNA nucleofected CAR T cells mediate effector function, their transient expression limits their applicability for solid tumours, where long-term immunosurveillance is likely to be required. To evaluate the effector function of the optimal CAR(2448)L construct in a long-term expression model, T cells were transduced lentivirally with the CAR(2448)L-BBz, CAR(2448)L-28z, CAR(αCD19)L-BBz, or CAR(αCD19)L-28z constructs (Figure 1b). Lentiviral transductions resulted in CAR surface expressions of: CAR(2448)L-BBz (34.7 ± 14.0%), CAR(2448)L-28z (57.7 ± 11.6%), CAR(αCD19)L-BBz (37.6 ± 15.2%), and CAR(αCD19)L-28z (51.1 ± 15.2%) (Figure S4).

**Figure 3:**
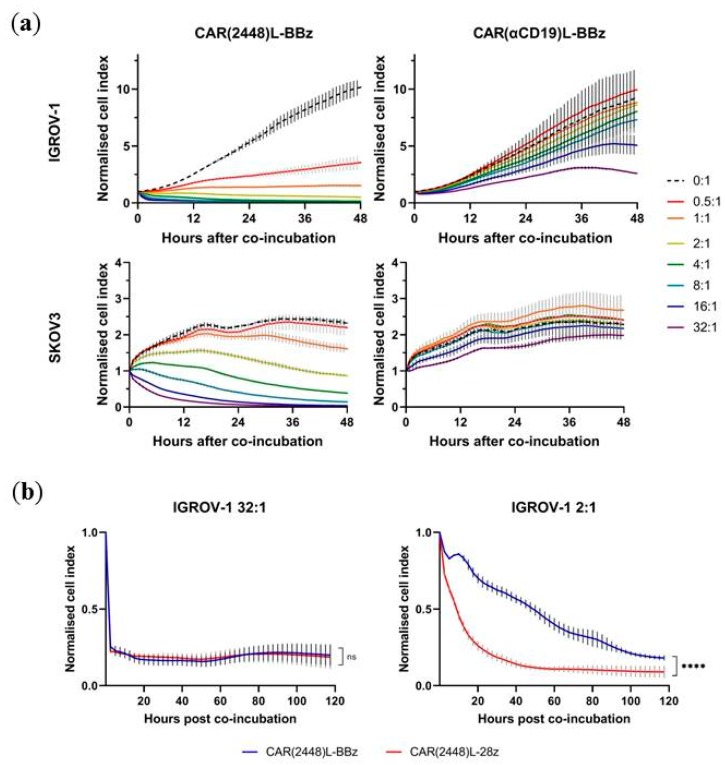
Lentivirally transduced CAR(2448)L T cells mediates dose-sensitive cytotoxicity upon recognition of target cells. (**a**) Real-time cytotoxicity of CAR(2448)L-BBz and control CAR(αCD19)L-BBz T cells against target cells at varying E:T ratios. Cytotoxic activity of CAR T cells only observable in CAR(2448)L T cells against ANXA2+ target cells. (**b**) Real-time cytotoxicity of CAR(2448)L T cells against IGROV-1 target cells at 32:1 and 2:1 E:T ratios. For all subfigures, abbreviations: ns not significant. **** *p* < 0.0001.

CAR T cells were subsequently co-incubated with target cells at varying E:T ratios. While CAR(2448)L-BBz T cells mediated cytotoxicity against target tumour cells even at low E:T ratios, control CAR(αCD19)L-BBz T cells were incapable of inducing cytotoxicity against target tumour cells even at E:T ratios as high as 32:1 (Figure 3a), thereby suggesting the sensitivity of CAR(2448)L to ANXA2+ cells. Similar results were also found for CAR(2448)L-28z when compared to CAR(αCD19)L-28z (Figure S5). Minimal cytotoxicity was observed against the ANXA2- HFF-1 cell line compared to control (Figure S6).

While there was no significant difference between the cytotoxicity of CAR(2448)L-BBz and CAR(2448)L-28z against IGROV-1 at the 32:1 E:T ratio, lower E:T ratios revealed a significant difference in cytotoxic activity between CD28 and 4-1BB containing CARs (Figure 3b). In addition, CAR(2448)L T cells tested against SKOV3 had significant differences in cytotoxicity between CD28 and 4-1BB at all E:T ratios tested (Figure S7). This suggests that the real-time in vitro assays are capable of differentiating the variance in activation profiles of CD28 and 4-1BB. 

**Figure 4:**
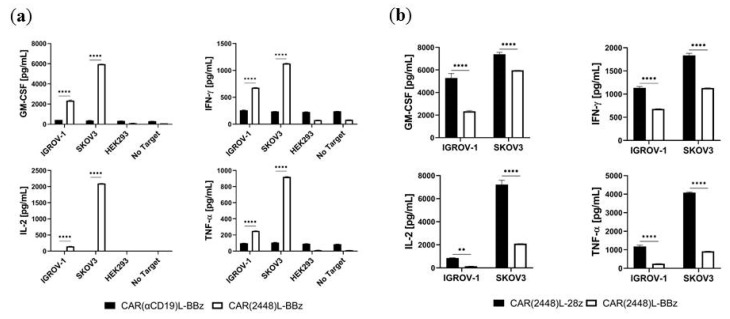
Lentivirally transduced CAR(2448)L T cells mediate cytokine release upon recognition of target cells. Co-incubation of CAR T cells and tumour cells at 10:1 E:T ratio. (**a**) CAR(2448)L-BBz T cells secrete inflammatory cytokines upon recognition of ANXA2+ target cells, but not control CAR(αCD19)L-BBz T cells. Cytokine release above basal level is not observed in co-incubation with ANXA2- cell lines. (**b**) CAR(2448)L-28z T cells secrete significantly higher levels of inflammatory cytokines compared with CAR(2448)L-BBz T cells upon recognition of ANXA2+ target cells. For all subfigures, abbreviations: ** *p* < 0.01. **** *p* < 0.0001.

The ability of CAR T cells to secrete inflammatory cytokines upon recognition of target cells is a key indicator of their anti-tumour activity [21]. CAR(2448)L-BBz T cells secreted an increased quantity of GM-CSF, IFN-γ, IL-2, and TNF-α compared to CAR(αCD19)L-BBz T cells upon co-incubation with the ANXA2+ cell lines IGROV-1 and SKOV3; but CAR T cells co-incubated with the control ANXA2- cell lines HEK293 and HFF-1 secreted only baseline levels of inflammatory cytokines (Figure 4a, Figure S8). Comparable results were also found for CAR(2448)L-28z T cells (Figure S9).

Of note, CAR(2448)L-28z T cells secreted significantly higher quantities of inflammatory cytokines compared to CAR(2448)L-BBz T cells (Figure 4b), consistent with the more rapid activation profile of CAR T cells with the CD28 costimulatory domain [22].

### 2.4. CAR(2448) T Cells Exhibit Anti-Tumour Activity in Vivo

Changing the costimulatory signal of CAR T cells can alter their metabolic profile. CAR T cells containing the CD28 costimulatory domain have increased glycolysis, while 4-1BB CAR T cells upregulate fatty acid oxidation pathways [23]. Hence, 4-1BB CAR T cells have a metabolic program more suited to surviving in the tumour microenvironment [24]. In addition, the significantly higher levels of inflammatory cytokines secreted by CAR(2448)L-28z compared to CAR(2448)L-BBz is of physiological concern, considering the effects of cytokine release in other CAR constructs [25]. As such, in vivo evaluation was carried out on CAR T cells containing the 4-1BB domain. 

To determine the function of CAR(2448)L in vivo, NIKO (NOD-scid IL-2Rγ-Knock-Out) mice were subcutaneously injected with SKOV-3 cells, similar to Cua et al. [11]. Tumours were allowed to grow to an average size of 100 mm^3^ before the mice were randomized and injected with CAR(2448)L-BBz or control CAR(αCD19)L-BBz T cells via intravenous tail vein infusion. Tumour growth was monitored twice a week by perpendicular calliper measurement.

**Figure 5:**
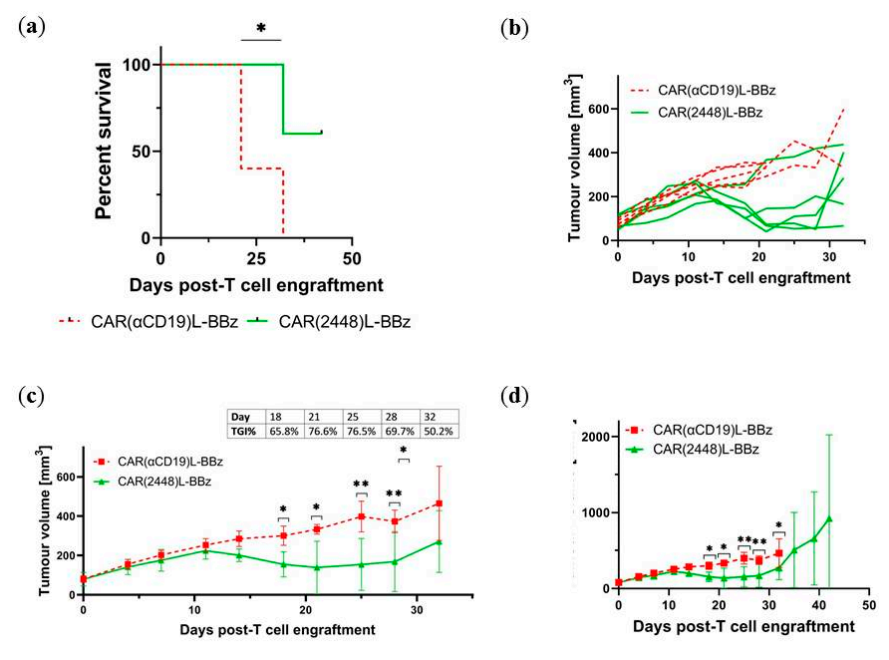
CAR(2448)L T cells mediate anti-tumour activity in vivo against ANXA2+ target cells. (**a**) Treatment with CAR(2448)L-BBz T cells confers a survival advantage on SKOV3 xenograft NIKO mice compared with control CAR(αCD19)L-BBz T cells. (**b**) CAR(2448)L-BBz T cells induce a reduction in tumour burden of SKOV3 xenograft NIKO mice compared to control CAR(αCD19)L-BBz T cells. (*n* = 5 per group) (**c**) Mice treated with CAR(2448)L-BBz T cells have significant reductions in mean tumour burdens compared with mice treated with CAR(αCD19)L-BBz T cells. Maximal tumour growth inhibition is observed 3 weeks after CAR(2448)L-BBz T cell treatment. (**d**) Tumours of mice treated with CAR(2448)L-BBz T cells subsequently relapse. For all subfigures, abbreviations: TGI%—tumour growth inhibition ratio. * *p* < 0.05. ** *p* < 0.01.

Mice in the CAR(αCD19)L-BBz control group had a median survival time of 21 days post T cell engraftment, while mice in the CAR(2448)L-BBz group survived significantly longer (median survival time not reached by day 42) (Figure 5a). While mice treated with control CAR(αCD19)L-BBz T cells died of increasing disease burden in a 3–4 week timespan, mice treated with CAR(2448)L-BBz T cells had observable decreases in tumour burden, with a maximum mean tumour growth inhibition of 76.6% on day 21 (Figure 5b,c), before subsequent tumour relapse (Figure 5d). Mice treated with control CAR T cells had minor weight loss compared with mice treated with CAR(2448)L-BBz (Figure S10).

**Figure 6:**
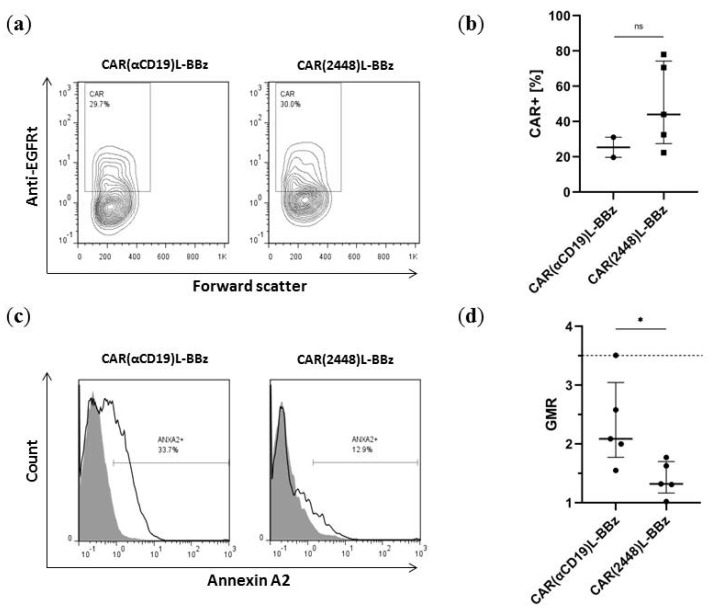
Control of ANXA2 in vivo (**a**) Representative flow cytometry of lymphocytes sampled from mice in endpoint assays. CAR T cell populations are still observed up to experimental endpoints. (**b**) Distribution of CAR T cell persistence in lymphocyte populations of NIKO mice engrafted with CAR T cells. No significant difference in CAR T cell persistence between CAR(αCD19)L-BBz and CAR(2448)L-BBz T cell infused mice. (**c**) Representative flow cytometry of endpoint tumours. Detection of ANXA2 via mAb 2448. (**d**) Distribution of ANXA2 expression in the tumours post CAR T cell treatment. Tumours of mice treated with CAR(2448)L-BBz T cells expressed significantly lower levels of ANXA2 than mice treated with control CAR(αCD19)L-BBz T cells. Dotted line represents geometric mean ratio of SKOV3 cells before injection into mice. For all subfigures, abbreviations: ns not significant. * *p* < 0.05. GMR—geometric mean ratio between 2448 binding and control.

Endpoint flow cytometry of blood samples from mice in this experiment reveal that CAR T cells persist in the bloodstream through the experimental duration, regardless of target (*p* = 0.191) (Figure 6a,b). However, while the tumours of mice treated with control CAR(αCD19)L-BBz T cells were ANXA2+, the expression of ANXA2 was greatly diminished in the tumours of mice treated with CAR(2448)L-BBz T cells (*p* < 0.05) (Figure 6c,d). In addition, reduced vasculature was observed in the tumours of mice treated with CAR(2448)L-BBz (unreported observation). These findings are consistent with the ANXA2 heterogeneity in SKOV3, with remission induced by CAR(2448)L, and subsequent relapse via the outgrowth of residual ANXA2- tumour cells.

## 3. Discussion

A limited number of viable targets preclude the broader use of CAR T cells in the treatment of cancer [26]. While more studies will be carried out, the results presented here suggest that CAR(2448) is potentially a good candidate for the treatment of ovarian cancers. CAR(2448) recognises an N-linked glycoepitope of ANXA2 that is expressed by ovarian cancer cell lines with an epithelial EMT phenotype. Aberrant glycosylation pathways in cancer generate glyco-neoepitopes that have the potential to be cancer-specific [6]. For example, alpha-fetoprotein has various glycoforms that are used in the detection of hepatocellular carcinoma and non-seminomatous germ cell tumours [27]. Such targets have the potential of reducing the risk of on-target, off-tumour activity. Nonetheless, the critical importance of target specificity in CAR T cells was highlighted by the case report of severe off-tumour effects in a patient treated with a trastuzumab-based CAR [28], which targets a glycoepitope of HER2 [29].

While mRNA transduced CAR T cells have transient expression of the CAR construct, making them infeasible in a therapeutic model where long-term immunosurveillance is required, the low cost and generation time of mRNA vectors compared to lentiviral vectors make them ideal for the purpose of evaluating the efficacy of CAR constructs in vitro. Here, we show that mRNA CAR vectors, in combination with real-time cytotoxicity assays, are capable of identifying optimal CAR constructs for cytotoxicity against target solid tumour cells.

In particular, the spacer length of CAR T cells is amenable to such in vitro optimisation. CAR(2448) was found to require a long spacer chain in order to induce optimal cytotoxicity. This is consistent with the ANXA2 target, which is known to be a membrane proximal glycoprotein [16]. The process of using mRNA CAR vectors to rapidly evaluate CAR constructs has potential for the drug development pipeline, as the efficacy of potential antibody CAR conversion candidates can be explored efficiently, even without further knowledge of the target antigen or the membrane proximity of the target epitope. In addition, this approach would allow the predeveloped mRNA vectors to be utilised to evaluate any safety concerns due to on-target, off-tumour activity in clinical trials [30].

Furthermore, the determination of the optimal spacer length for a particular CAR was previously only observable in in vivo xenograft models [20] and suggests the potential role of real-time cell growth monitoring assays for the purpose of CAR optimisation in vitro.

CAR(2448) induces specific anti-tumour functions upon recognition of target ovarian cancer cells expressing ANXA2 in vitro, inducing inflammatory cytokine secretion and potent cytotoxicity against target cells.

While the secretion of inflammatory cytokine is essential for T cell cytotoxicity against tumour cells, the production of GM-CSF may be of concern, as the stimulation of macrophages has been found to contribute to the neurotoxicity observed in anti-CD19 CAR T cell therapy. As such, GM-CSF knockout may be necessary in order to improve the safety profile of CAR(2448) [31].

Antigen negative relapse is a known outcome of CAR T cell therapy [32]. Tumour heterogeneity causes the outgrowth of residual antigen negative tumour cells upon the elimination of antigen positive target cells. For the xenograft experiments reported here, SKOV3 was used as a model of heterogeneous ANXA2 expression. In this case, the target ANXA2 is known to promote neovasculature via plasmin generation [17]. As such, the depletion of ANXA2+ cells from the tumour could potentially inhibit tumour growth via the removal of an angiogenic pathway. We observed reduced vasculature in the tumours of mice treated with CAR(2448) (data not shown), and it would be worth exploring this as a route by which CAR(2448) effects its anti-tumour function. The use of immunohistochemistry to detect the presence of CAR T cells, vasculature, and ANXA2+ cells in the tumour would clarify the function of CAR(2448) in vivo.

To further explore CAR(2448) as a drug candidate, it would be interesting to explore the persistence of CAR(2448) T cell in the various compartments of the xenograft model. The exhaustion status of CAR(2448) T cells in the intratumoural space, and the formation of a memory T cell niche, will both contribute to understanding the function of CAR(2448).

Nonetheless, the evidence of antigen-negative relapse presented by the xenograft model here suggests the need for further development of CAR(2448) as a therapeutic. In addition, the hostile tumour microenvironment is likely to be more challenging to treat than the xenograft model used here as proof-of-concept. Immunosuppressory cells, immune checkpoint molecules, and immunosuppressive cytokines all contribute to limiting the efficacy of CAR T cells in solid tumours [33].

It is possible that further engineering of CAR(2448) T cells to deliver a therapeutic payload such as IL-12 or IL-18 upon recognition of target cells will be sufficient for tumour clearance, via the remodelling of the tumour microenvironment and the recruitment of innate immune cell populations to the tumour site [34].

Alternatively, CAR(2448) could be used in combination with other orthogonal therapeutics against epithelial ovarian cancer. A number of CARs against various ovarian cancer targets have been in development, and it is possible that the combination of CARs against separate antigen targets will result in an effective therapy [35]. While the use of checkpoint inhibitors for the treatment of ovarian cancer has not been found to be effective [36], it is possible that they may function well in combination with CAR(2448).

While radiotherapy is rarely used in the treatment of ovarian cancer [37], recent evidence suggests that radiotherapy may be an effective preconditioning regimen for CAR T cell therapy [38]. In addition to promoting T cell infiltration into the tumour, radiation also upregulates the expression of cell death ligands such as Fas and TRAIL that induce T cell cytotoxicity against tumour cells [38,39]. 

In summary, CAR(2448) targets a glycosylated epitope of ANXA2, which was previously shown to be expressed across various epithelial ovarian cancer cell lines. CAR(2448) T cells induce inflammatory cytokine release and cytotoxicity against tumour cells expressing ANXA2 *in vitro.* CAR(2448) was also found to prolong survival and reduce ovarian tumour burdens in a xenograft model. CAR(2448) is hence a potential candidate worth further exploration for the treatment of ovarian cancer.

## 4. Materials and Methods

### 4.1. Cell Lines and Culture

Ovarian cancer cell lines were a kind gift from Dr. Ruby Huang (Cancer Science Institute, Singapore). HEK293, HFF1, and IMR90 were purchased from the ATCC. IGROV-1 was cultured in RPMI-1640 with 10% FBS. SKOV3, was cultured in a 1:1 ratio of DMEM (high glucose) and DMEM (low glucose) with 10% FBS added. IMR90, HEK293, and HFF-1 were cultured in DMEM (high glucose) supplemented with 10% FBS and 2 mM L-Glutamine.

Peripheral blood mononuclear cells (PBMCs) were purchased from StemCell Technologies. T cells were isolated from PBMCs using the EasySep™ Human T Cell Isolation Kit (StemCell Technologies, Vancouver, BC, Canada), according to the manufacturer’s protocol. In standard culture, T cells were activated with anti-CD3/CD28 Dynabeads (Life Technologies, Grand Island, NY, USA) at a 1:1 bead-to-cell ratio, and cultured in RPMI-1640 supplemented with 10% FBS, and a cytokine cocktail of IL-7 (20 U/mL), IL-15 (10 U/mL), and IL-21 (0.04 U/mL).

### 4.2. mRNA In Vitro Transcription and Nucleofection of T Cells

The CAR(2448) constructs consist of the V_L_ and V_H_ chain sequences from mAb 2448 upstream of the IgG4 hinge and Fc region (where mentioned, the IgG4 linker was replaced by truncated variants), followed by the CD28 transmembrane and intracellular signalling domains, and the CD3ζ signalling domain. The eGFP or EGFRt reporters were connected downstream of the construct using a T2A linker. The whole construct was cloned into a pcDNA3.1(+) vector backbone (GenScript). 

Linearised templates were generated via XbaI restriction digestion, and CAR(2448) mRNA was in vitro transcribed using the HiScribe™ T7 ARCA mRNA Kit (with tailing) (NEB), following the manufacturer’s instructions. Synthesised ARCA capped and poly(A)-tailed mRNA was quantified on a Nanodrop ND-1000 spectrophotometer, and stored in single-use aliquots at –80 °C.

Nucleofection of mRNA into T cells was carried out on a 4D-Nucleofector system with the P3 Primary Cell 4D-Nucleofector^®®^ X Kit according to the manufacturer’s protocol. Briefly, 1×10^6^ or 5×10^6^ T cells were mixed with equimolar concentrations of mRNA before nucleofection using program EO-115. Nucleofected T cells were immediately utilised in functional assays.

### 4.3. Lentiviral Transduction of T Cells

Third-generation lentiviral constructs were commercially synthesised (VectorBuilder, Chicago, IL, USA). For lentiviral transduction, T cells were cultured in serum-free TexMACS™ Medium (Miltenyi Biotec, Bergisch Gladbach, Germany) supplemented with a cytokine cocktail of IL-7 (20 U/mL), IL-15 (10 U/mL), and IL-21 (0.04 U/mL). Three days before transduction, T cells were isolated and activated at a 3:1 bead-to-cell ratio. Non-treated, sterile 24-well plates were coated with retronectin (25 µg/mL), and incubated for 2 h at room temperature, or at 4 °C overnight. Coated plates were blocked with 2% BSA/PBS for 30 min, washed with PBS, and stored at 4 °C before use.

Lentiviral CAR vectors (corresponding to a multiplicity of infection of 10) were added to each well containing 1×10^6^ T cells (for a total volume of 0.5 mL) before spinoculation for 2 h at 1200 g, 32 °C. Transduced T cells were incubated at 37 °C, 5% CO2 for 16 h before a second round of transduction was conducted. CAR-transduced T cells were the cultured in standard culture.

### 4.4. Flow Cytometry Analysis

The MACSQuant Analyser X was used for flow cytometric analysis. Live cell populations were gated on propidium iodide-negative populations. CAR T cells with the EGFRt reporter gene were incubated with cetuximab for 45 min at 4 °C, washed, and incubated with fluorescein isothiocyanate-labelled goat anti-human kappa light chain mAbs for 15 min at 4 °C. CAR T cells with the eGFP reporter gene were measure directly. Flow cytometry data was analysed on FlowJo 7.6.5, with cell debris gated out on forward scatter (FSC-H) and side scatter (SSC-H). Fluorescence positive populations were gated based on negative control and isotype control samples.

### 4.5. Cytotoxicity Assays

For the real-time monitoring of target cell growth, 5×10^3^ target cells were seeded per well in an E-Plate 96 (ACEA Biosciences, San Diego, CA, USA). Growth was monitored in the xCELLigence RCPA MP Instrument for 24 h before the addition of CAR T cells at varying effector-to-target ratios. Target cell growth was monitored on the xCELLigence system (ACEA Biosciences, San Diego, CA, USA) for another 96 h. Cell indexes were normalised to the co-incubation time-point.

### 4.6. Cytokine Release Assay

CAR T cells were co-incubated with target cells at a 10:1 effector-to-target ratio for 6 h at 37 °C, 5% CO2. To analyse T cell cytokine secretion, the MACSPlex Cytokine 12 kit (Miltenyi Biotec, Bergisch Gladbach, Germany) was utilised according to the manufacturer’s protocol. Briefly, supernatants from the co-incubations were collected, and cytokines in the supernatants were captured by MACSPlex capture beads, before labelling with APC-conjugated antibodies corresponding to each cytokine. A standard curve was used to calculate the concentration of each cytokine on the MACSQuant Analyzer X.

### 4.7. Xenograft Murine Model

NIKO mice were generated by knocking-out IL-2R gamma chain with CRISPR/cas9 technology in NOD-scid mice in the Biological Resource Centre, Agency for Science, Technology and Research (A*STAR), Singapore. Eight week old female NIKO mice were administered 2 × 10^6^ SKOV3 cells (in 200 µL 1:1 media:matrigel) via subcutaneous injection into the right flank. 5 × 10^6^ CAR T cells were administered via tail vein injection after tumours reached an average volume of 100 mm^3^ ± 20 mm^3^. Tumour volumes were calculated via the formula: Tumour volume = (width × width × length)/2. Mice were culled when their tumour volume exceeded 2000 mm^3^, when the tumour site was severely ulcerated, or when severe, persistent weight loss was observed. The tumour growth inhibition ration (TGI%) was calculated as

$$TGI{\%}_{day\;n}=\frac{C_{day\;n}-T_{day\;n}}{C_{day\;n}-C_{day\;0}}\times 100\%$$

where: C=Mean control tumour volume. T=Mean treated tumour volume

Animals were handled according to A*STAR (BRC) IACUC Protocol No.:151001, in accordance with the National Advisory Committee for Laboratory Animal Research (NACLAR) Guidelines. 

### 4.8. Statistical Analysis

Data was analysed using Excel (Microsoft) and Prism 8 (GraphPad, San Diego, CA, USA). Two-tailed unpaired t tests we used for the comparison of two groups. Multiple groups were compared by one-way ANOVA, with multiple comparisons. Survival data was analysed using the log-rank test. The Mann–Whitney test was used for tumour analysis.

## Supplementary Materials

Supplementary materials can be found at https://www.mdpi.com/1422-0067/21/2/381/s1.

## Abbreviations

CAR	Chimeric antigen receptor
OC	Ovarian cancer
mAb	Monoclonal antibody
ANXA2	Annexin A2
EMT	Epithelial-mesenchymal transition
E:T	Effector-to-target
PBMC	Peripheral blood mononuclear cell
NIKO	NOD-scid IL-2Rγ-Knock-Out
