# Supplementary material for: Preclinical Activity of Embryonic Annexin A2-Specific Chimeric Antigen Receptor T Cells Against Ovarian Cancer
Source: Int J Mol Sci. 2020 Jan 7;21(2):381. doi: 10.3390/ijms21020381 (PMC7013580; doi:10.3390/ijms21020381)
Supplement: Supplementary file 1 [file ijms-21-00381-s001.pdf]

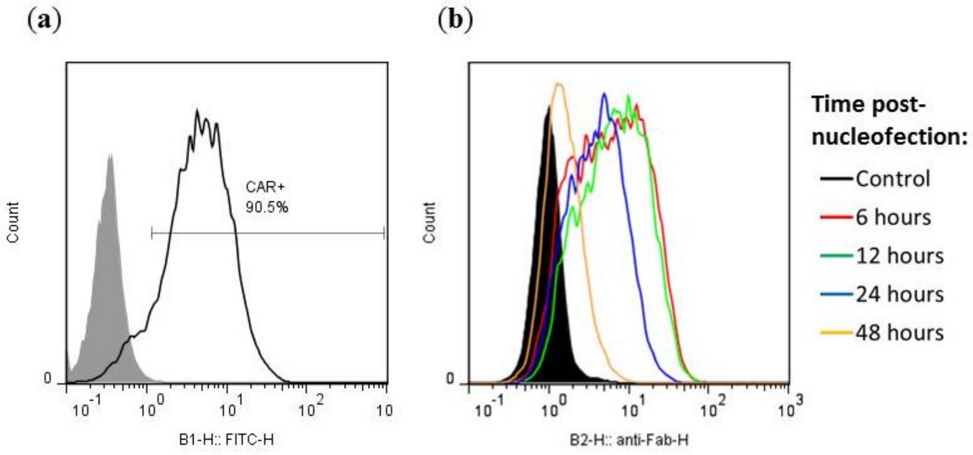

Figure S1. Expression profile of T cells nucleofected with CAR(2448) mRNA. (**a**) Representative flow cytometry of CAR(2448) expression on T cells, detected via eGFP reporter. (**b**) Expression of CAR(2448) on T cells over a 48 hour timeframe.

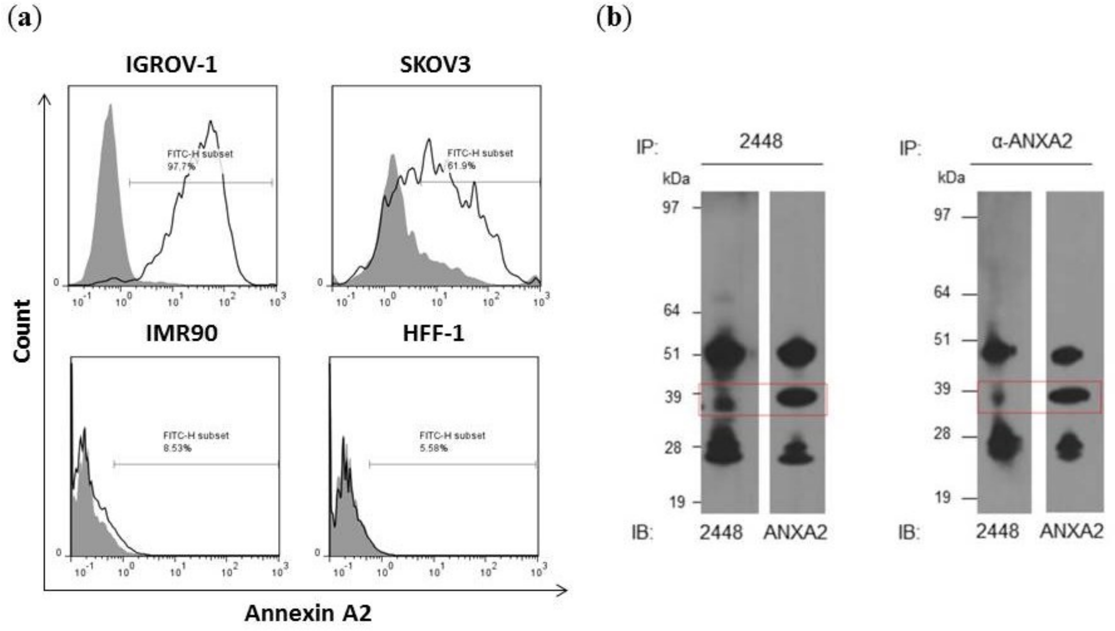

Figure S2. Expression of target antigen, annexin A2, on target cell lines. Detection of ANXA2 via mAb 2448. (**a**) Flow cytometry detection of surface ANXA2 expression. IGROV-1 is a highly ANXA2 positive cell line, while SKOV3 exhibits the heterogeneous expression of ANXA2. IMR90 and HFF-1 are ANXA2 negative. (**b**) Immunoprecipitation of IGROV-1 lysate with mAb 2448 (left panel) and commercial α-ANXA2 antibody (right panel). Cross probing with the different mAbs produce bands of a similar size (red box).

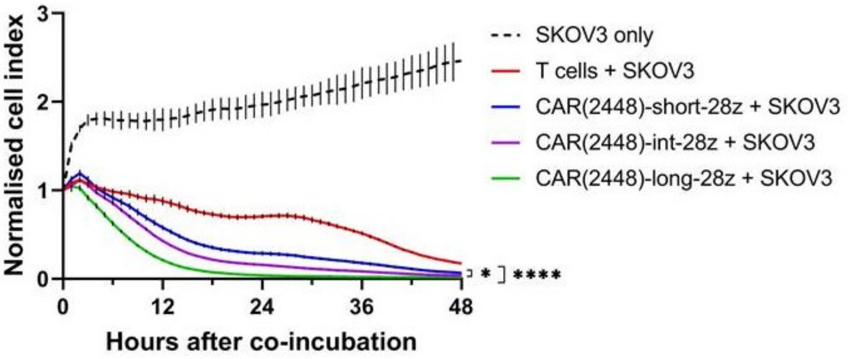

Figure S3. mRNA transfected CAR(2448) T cells mediate cytotoxicity. CAR(2448)-long-28z T cells mediate superior cytotoxicity against SKOV3 cells compared to CAR(2448)-short-28z or CAR(2448)-int-28z T cells.

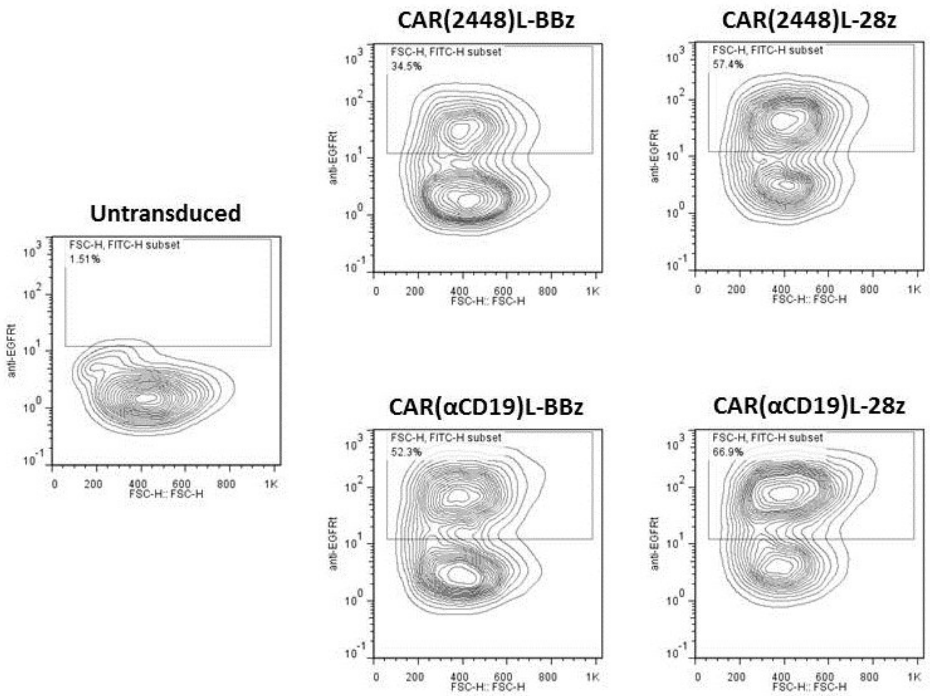

Figure S4. Expression of CAR(2448) by lentivirally-transduced T cells. Representative expression of CAR constructs on T cells, detected via cetuximab on EGFRt reporter gene.

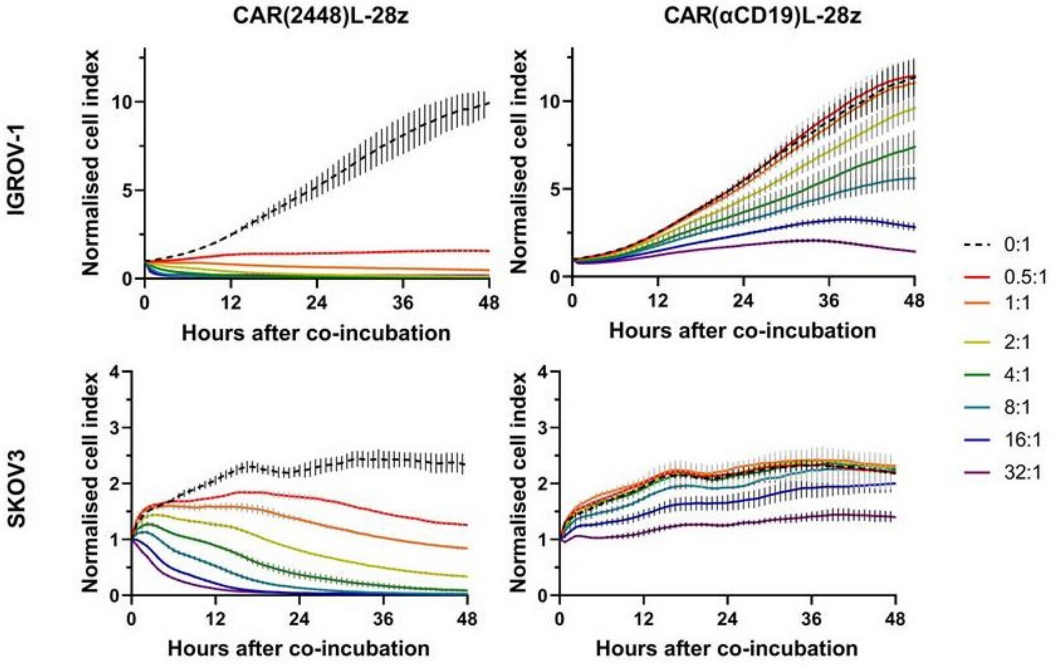

Figure S5. Cytotoxicity of CAR(2448)L-28z. Real-time cytotoxicity of CAR(2448)L-28z and control CAR(αCD19)L-28z T cells against target cells at varying E:T ratios.

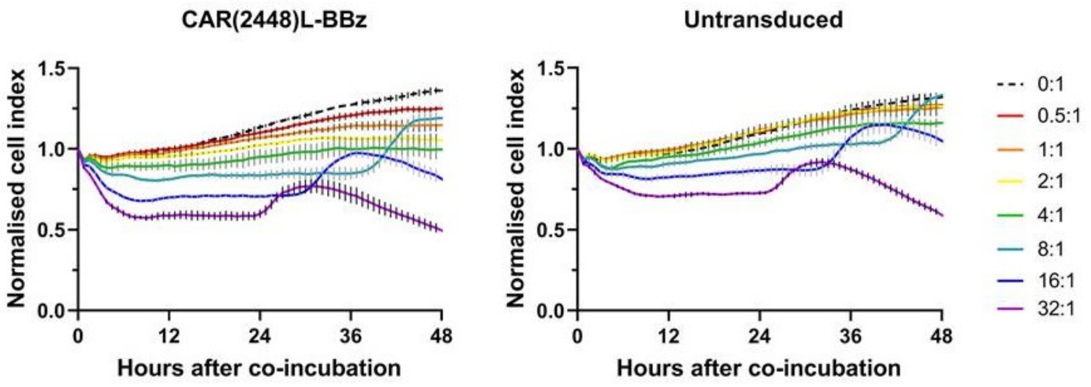

Figure S6. Cytotoxicity of CAR(2448)L-BBz against HFF-1. Real-time cytotoxicity of CAR(2448)L-BBz and control untransduced T cells against ANXA2- HFF-1 cells at varying E:T ratios. Cytotoxic activity of CAR T cells not observable in CAR(2448)L T cells against ANXA2- normal cells

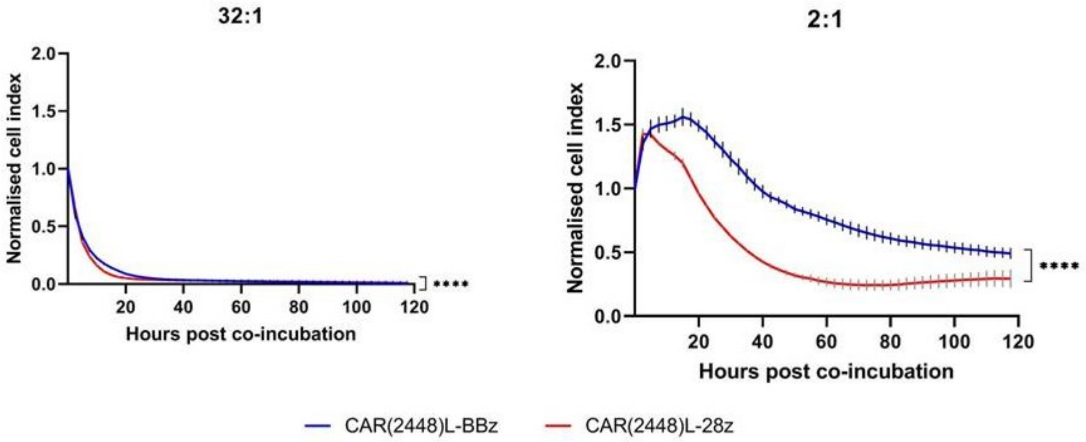

Figure S7. Lentivirally transduced CAR(2448)L T cells mediates dose-sensitive cytotoxicity upon recognition of target cells. Real-time cytotoxicity of CAR(2448)L T cells against SKOV3 target cells at 32:1 and 2:1 E:T ratios.

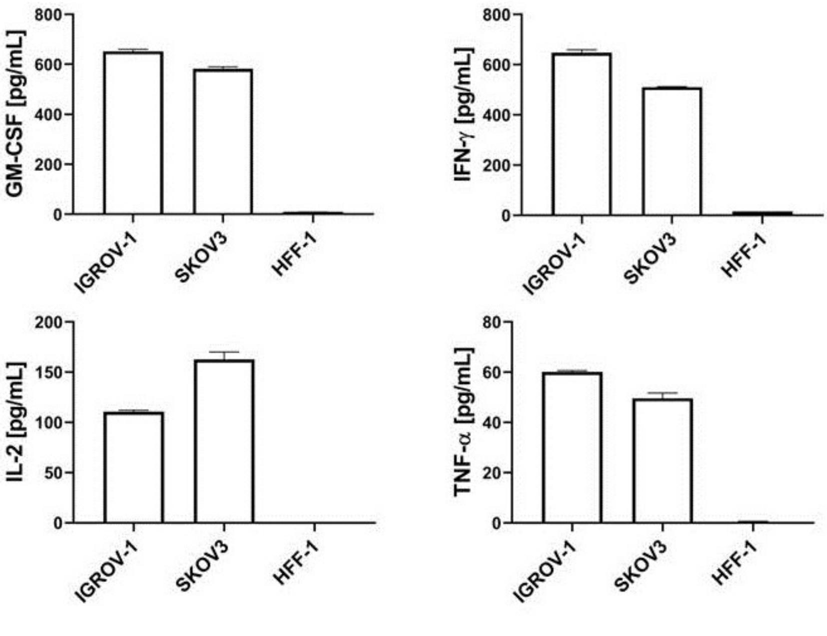

Figure S8. Cytokine release of CAR(2448)L-BBz. CAR(2448)L-BBz T cells secrete inflammatory cytokines upon recognition of ANXA2+ target cells, but not against the ANXA2- cell line HFF-1.

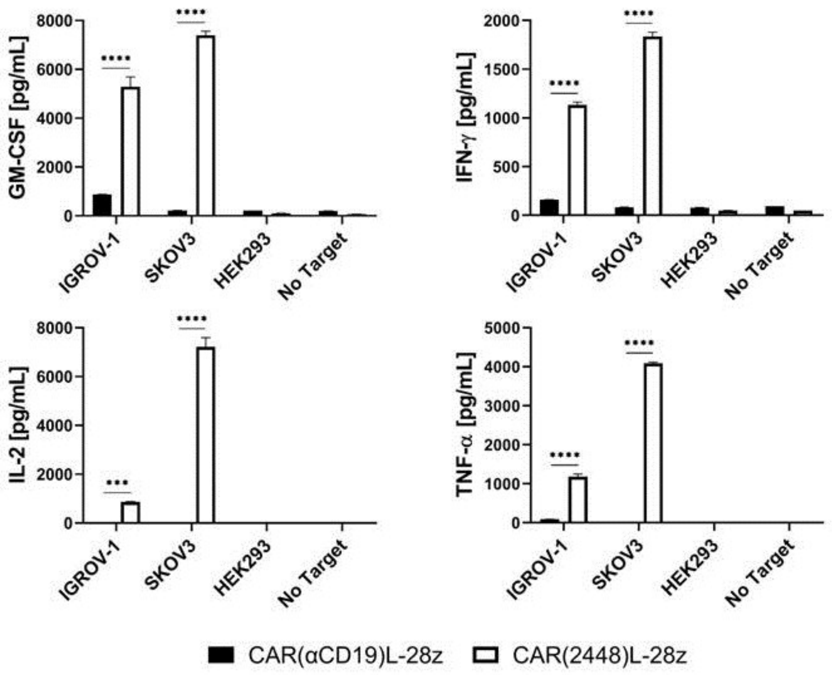

Figure S9. Cytokine release of CAR(2448)L-28z. CAR(2448)L-28z T cells secrete inflammatory cytokines upon recognition of ANXA2+ target cells, but not control CAR(αCD19)L-28z T cells. Cytokine release above basal level is not observed in co-incubation with ANXA2- cell lines.

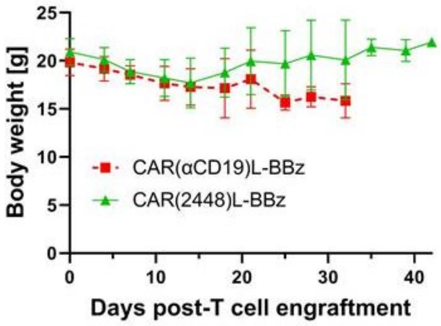

Figure S10. Body weight of mice treated with CAR T cells. Mice treated with the control CAR(αCD19)L-BBz exhibited minor weight loss compare to mice treated with CAR(2448)L-BBz as tumours progressed.
